# Jejunal Submucosal Hemangioma as a Cause of Massive Gastrointestinal Bleeding: A Case Report

**DOI:** 10.7759/cureus.8372

**Published:** 2020-05-31

**Authors:** Laith Numan, Ahmed Elkafrawy, Tim Brotherton, Andrew Tomaw, Donald Campbell

**Affiliations:** 1 Internal Medicine, University of Missouri-Kansas City School of Medicine, Kansas City, USA; 2 Gastroenterology, University of Missouri Kansas City and Saint Luke’s Hospital, Kansas City, USA

**Keywords:** gastrointestinal bleeding, hemangioma, anemia, git endoscopy

## Abstract

Small intestinal hemangiomas are uncommon tumors that frequently present with gastrointestinal bleeding (GIB). Diagnosis, detection, and treatment can be challenging and may require surgical intervention. An 81-year-old female presented with melena. Video capsule endoscopy revealed active bleeding in the proximal jejunum and push enteroscopy identified a polypoid nodule with central umbilication. The patient underwent laparoscopic resection and jejunal submucosal hemangioma was detected. Submucosal hemangiomas are a rare cause of GIB. As the most common site of submucosal hemangiomas is the mid-jejunum, they are not easy to detect. Surgical intervention is usually required for a definitive diagnosis and definitive treatment.

## Introduction

Intestinal hemangiomas are uncommon tumors, comprising only 0.05% of all intestinal neoplasms [1]. These benign vascular tumors may be multiple or solitary and can involve any segment of the gastrointestinal (GI) tract. However, the jejunum is the most common site of involvement with intestinal hemangiomas representing 5%-10% of all benign neoplasms of the small intestine [2]. Clinical manifestations of intestinal hemangioma are variable, including GI bleeding, intussusception, small bowel obstruction, and perforation [1, 3]. GI bleeding is the most frequent manifestation and may present with acute blood loss or occult bleeding and chronic anemia. 

The diagnosis of intestinal hemangioma can be challenging due to their location, and often no preoperative diagnosis is identified. Endoscopic resection is feasible for small lesions. However, surgical resection is the preferred treatment modality for large lesions or small lesions not amenable to endoscopic treatment. A case of an 81-year-old female with a jejunal hemangioma that was identified by capsule endoscopy and push enteroscopy before undergoing surgical resection is presented herein.

## Case presentation

An 81-year-old female presented with a one-week history of melena. The development of melena was associated with fatigue, pallor, palpitations, and shortness of breath on exertion. Hematemesis, hematochezia, nonsteroidal anti-inflammatory drug (NSAID) use, use of anticoagulants or antiplatelet agents were denied. Presenting blood pressure was 130/70 mmHg and the heart rate was 106 beats/minute. Her physical examination was unremarkable except for pallor and lower limb edema. 

**Figure 1:**
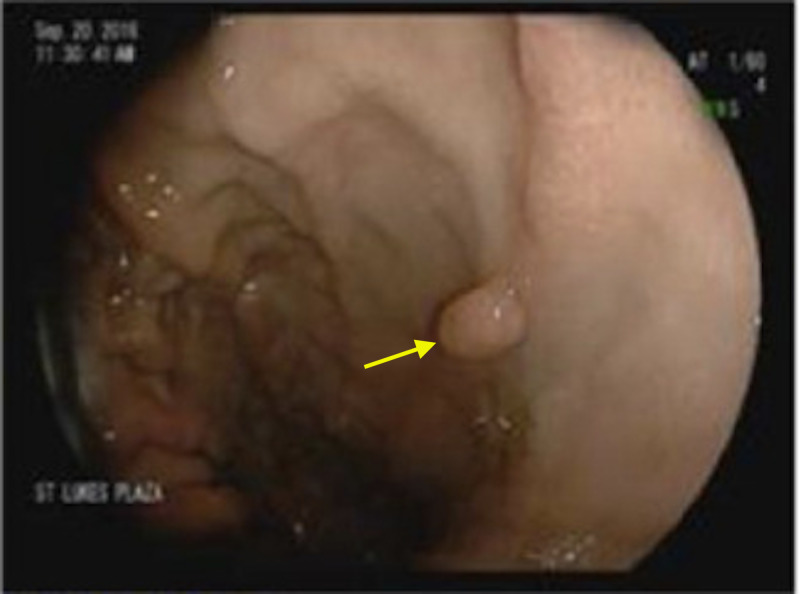
EGD image of the stomach body showing a benign polyp (arrow). EGD, esophagogastroduodenoscopy

Admission hemoglobin was 4.7 g/dL, which was lower than her baseline of 8 g/dL. Transfusions were administered to maintain hemoglobin above 7 g/dL, and a proton pump inhibitor infusion was initiated. Esophagogastroduodenoscopy (EGD) was performed; benign-appearing and non-bleeding polyps were detected in the gastric fundus and body (Figure 1).

**Figure 2:**
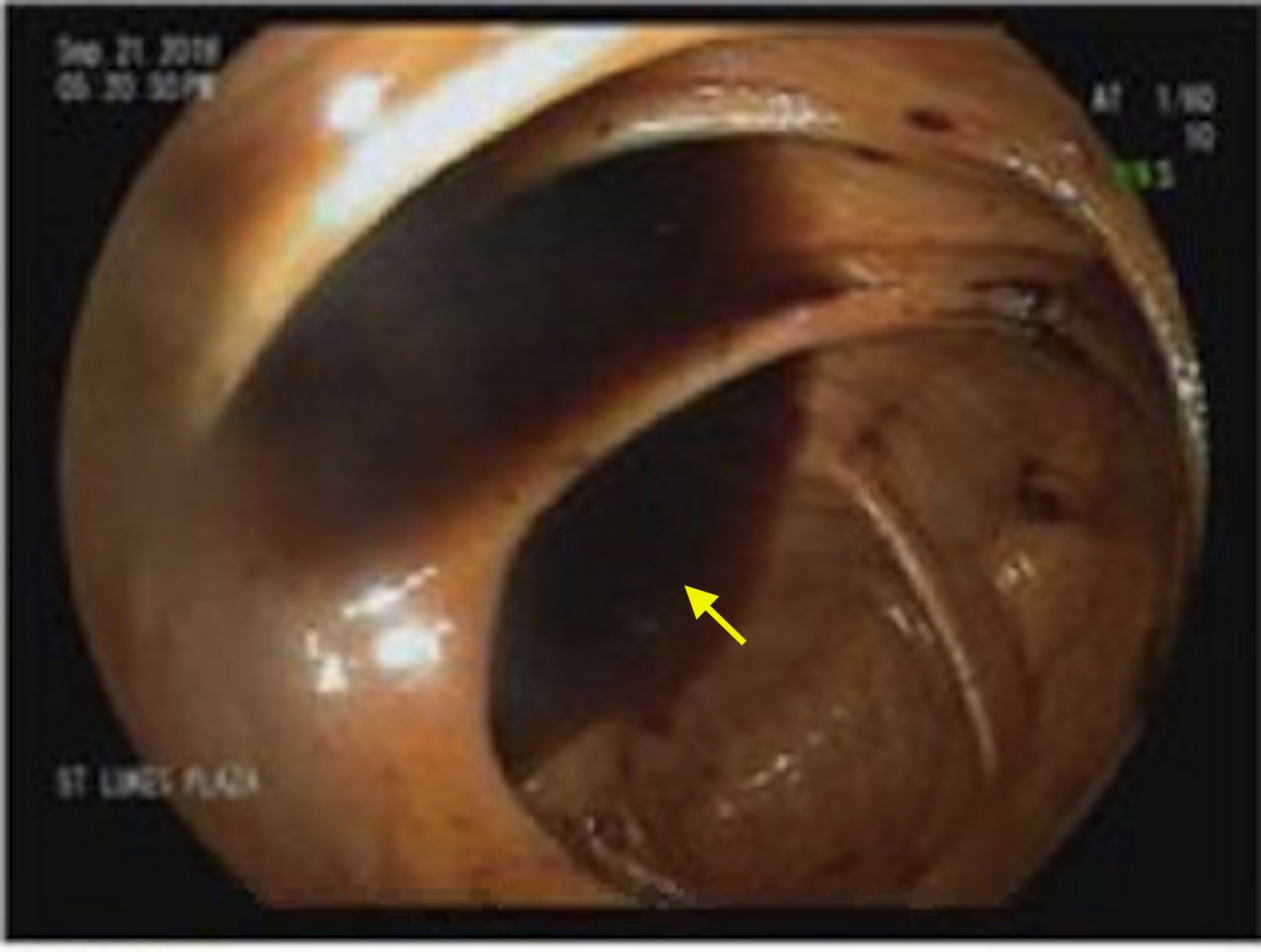
Colonoscopy image of the cecum showing melenic liquid (arrow).

The patient’s hemoglobin continued to trend downward, and melena continued. Subsequently, colonoscopy did not detect significant abnormalities other than moderate diverticulosis, internal hemorrhoids, and old melenic liquid throughout the colon and ileum but no active bleeding (Figure 2). 

**Figure 3:**
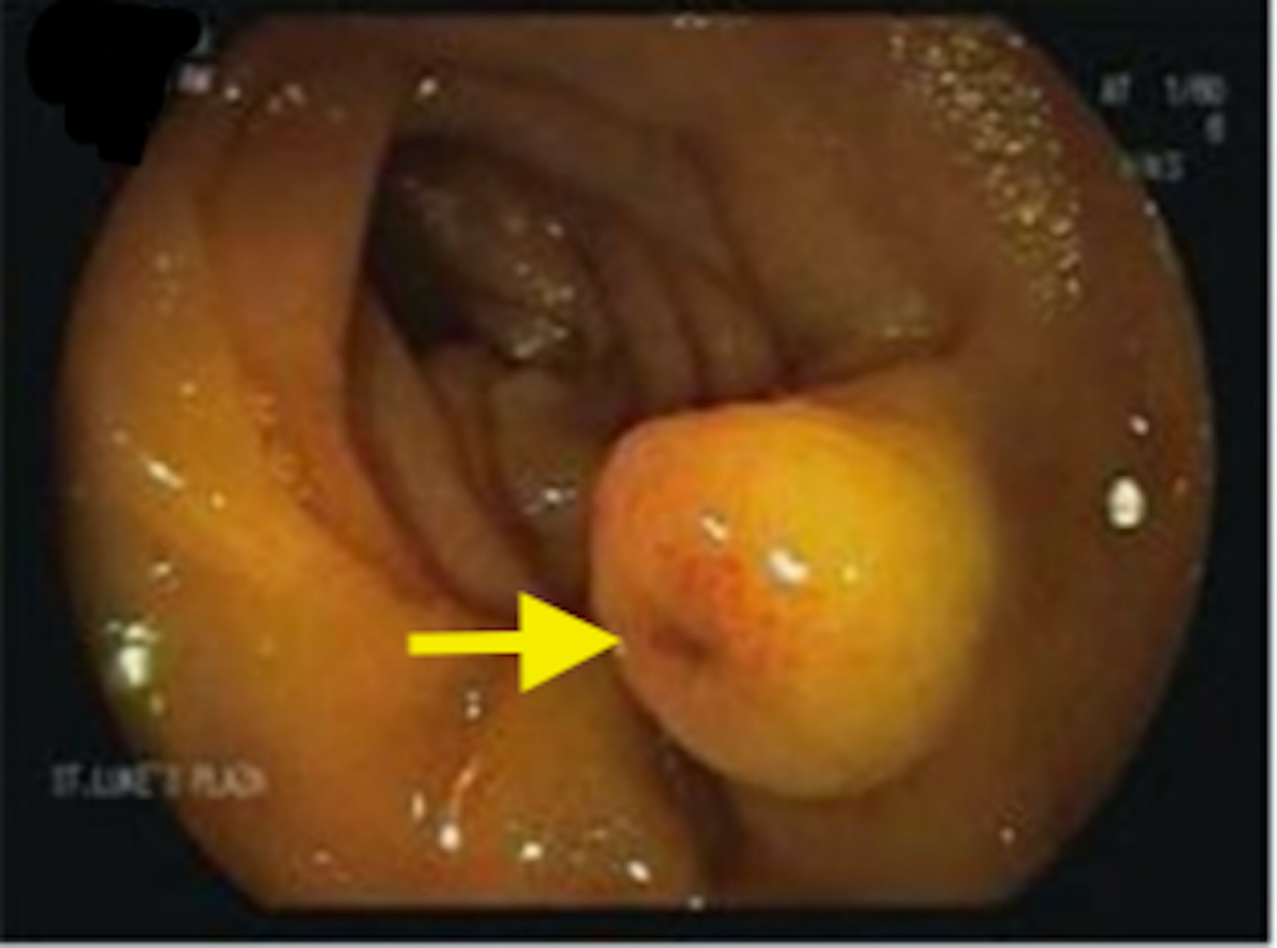
Push enteroscopy image of the polypoid lesion with central umbilication (arrow).

Due to ongoing transfusion requirements, a video capsule endoscopy was performed, and active bleeding was identified shortly after the capsule passed the duodenum. Push enteroscopy was then performed, and a polypoid nodule with central umbilication and red spot was detected in the proximal jejunum (Figure 3). 

**Figure 4:**
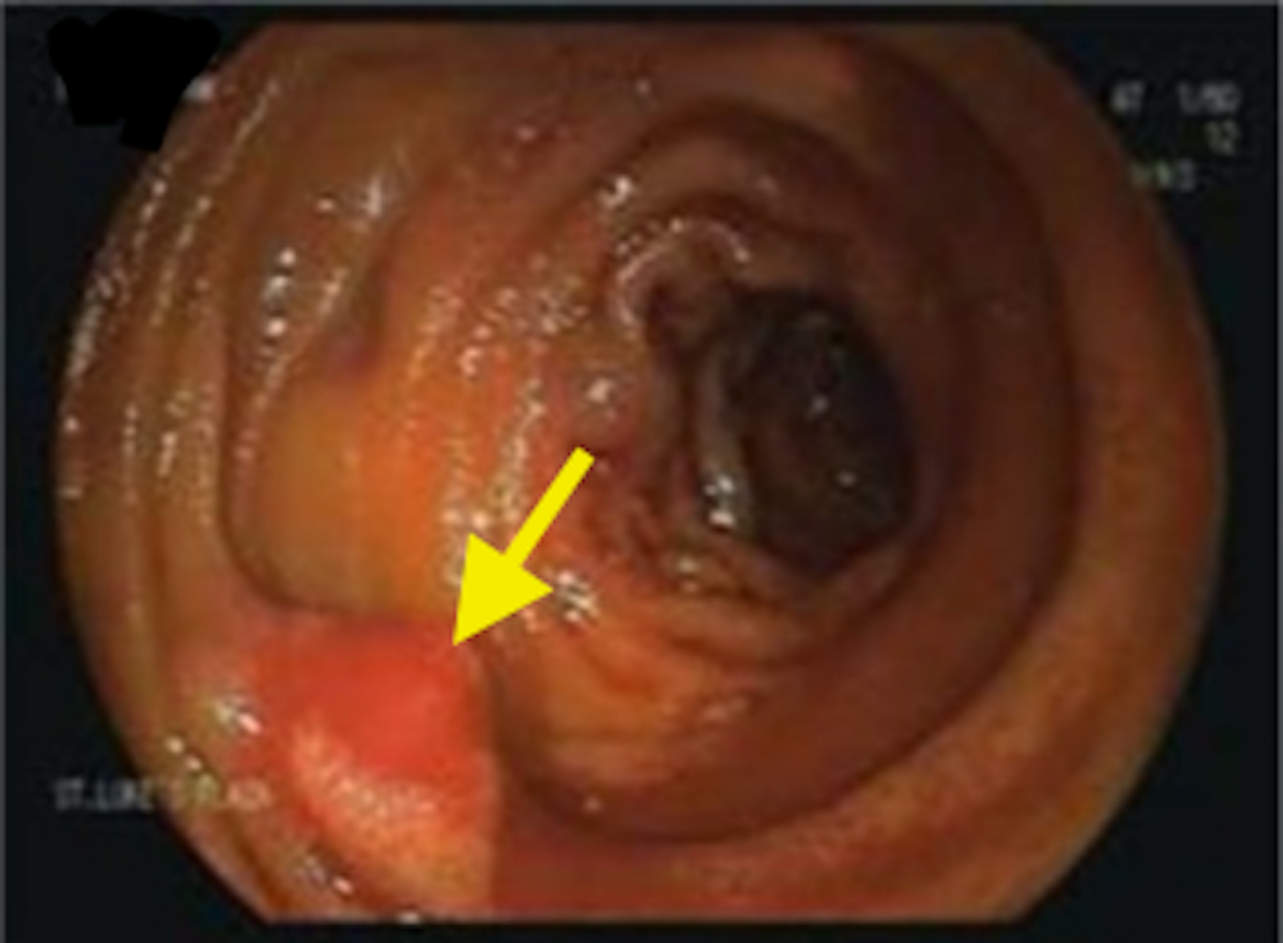
Image shows the polypoid lesion bleeding post-resection (arrow).

The lesion was biopsied and post-biopsy bleeding developed (Figure 4).

Hemostasis was achieved by injection of epinephrine (1:10000 concentration) and application of three hemostatic clips. After endoscopic intervention, the patient’s hemoglobin remained stable, and further transfusions were not required.

Due to concerns for malignancy and further bleeding a laparoscopic segmental jejunal resection was performed. Histological examination of the resected specimen confirmed a jejunal submucosal hemangioma with surrounding hemorrhage, an organizing hematoma, and no evidence of dysplasia or malignancy. The hemoglobin remained stable after surgery, and the patient was discharged home. On follow up, her hemoglobin normalized, her fatigue improved, and her melena resolved. 

## Discussion

Intestinal hemangiomas are rare tumors and they are therefore an uncommon cause of GI bleeding. Unlike arteriovenous malformations, hemangiomas are neoplastic lesions secondary to endothelial hyperplasia [4]. They may occur sporadically or as a part of several genetic syndromes, including Von-Hippel-Lindau and Maffucci syndromes [4]. Hemangiomas typically occur in younger patients, particularly in the third decade of life [5]. However, they can occur in any age group, such as in the 81-year-old patient reported herein. These tumors typically manifest as pedunculated masses found within the lumen of the small bowel. Less commonly, they may display an infiltrative growth pattern with extension into the submucosa and even muscularis layer [6].

The presentation of intestinal hemangioma is variable, with the most common presenting symptom being GI bleeding and anemia [7-8]. Bleeding may be occult or clinically evident, such as in this case with frank melena, severe anemia, and orthostatic symptoms. Less commonly, intestinal hemangiomas may cause abdominal pain, bowel obstruction, intussusception, and even perforation [9-10]. The physical exam is often unremarkable, as was the case in the patient described who had a normal abdominal exam. 

The diagnosis of intestinal hemangiomas is challenging as no imaging study can provide a definitive diagnosis. Due to their location in the small intestine, they are often not detected during upper endoscopy or colonoscopy. The advent of newer endoscopic techniques such as capsule endoscopy, antegrade and retrograde single and double-balloon enteroscopy, and push enteroscopy have allowed for enhanced detection of these lesions [11]. While the patient described was not definitively diagnosed until histological examination of the surgical specimen, capsule endoscopy and push enteroscopy were used to provide initial bleeding control and to identify the etiology and location of her GI hemorrhage. 

Push enteroscopy, unlike capsule endoscopy, also plays a role in the management of intestinal hemangioma, offering an opportunity for biopsy, hemostatic intervention, and definitive treatment [12-13]. As occurred in this case, biopsy carries a risk of bleeding. Furthermore, endoscopic snare resection is associated with a risk of incomplete resection as tumor depth of invasion can be challenging to predict [14]. Surgical resection of the hemangioma and affected segment of bowel is the preferred treatment as it is diagnostic and also offers definitive treatment of the lesion [10, 15]. While intestinal hemangiomas are a rare cause of GI hemorrhage, they can present with massive blood loss and surgical emergencies including obstruction, volvulus, and perforation.

This case was presented as a poster at the American College of Gastroenterology annual meeting [16].

## Conclusions

Submucosal hemangiomas are a rare cause of GIB. They can present with melena and severe anemia. As the most common site is mid-jejunum, it is difficult to detect submucosal hemangiomas. In this case, the lesion was detected by video capsule endoscopy and was reached by push enteroscopy. Surgery is usually required for a definitive diagnosis and for definitive treatment.
